# The impact of PD-1 inhibitors on prognosis in unresectable hepatocellular carcinoma treated with TACE and lenvatinib: a retrospective study

**DOI:** 10.1038/s41598-024-63571-1

**Published:** 2024-06-21

**Authors:** Zongren Ding, Guoxu Fang, Yanyan Tang, Yongyi Zeng

**Affiliations:** 1 The First Affiliated Hospital of Fujian Medical University, Fuzhou, 350001 China; 2 Department of Hepatopancreatobiliary Surgery, Mengchao Hepatobiliary Hospital of Fujian Medical University, Fuzhou, 350001 China; 3 Fujian Provincial Liver Disease Research Center, Fuzhou, 350001 China; 4 Department of Radiology, Mengchao Hepatobiliary Hospital of Fujian Medical University, Fuzhou, 350001 China

**Keywords:** Hepatocellular carcinoma, Transarterial chemoembolization, Lenvatinib, PD-1 inhibitors, Cancer, Cancer therapy

## Abstract

Our aim was to explore whether programmed death receptor-1 (PD-1) inhibitors would improve the prognosis of unresectable hepatocellular carcinoma (HCC) treated with transarterial chemoembolization (TACE) plus lenvatinib. In this single-center retrospective study, patients with unresectable HCC who underwent TACE and were administered lenvatinib with or without PD-1 inhibitors were enrolled and divided into the TACE + lenvatinib group and TACE + lenvatinib + PD-1 group. Overall survival (OS), progression-free survival (PFS) and tumor response were assessed by the Response Evaluation Criteria in Solid Tumors (RECIST v1.1 and mRECIST). Treatment-related adverse events (AEs) were evaluated according to the Common Terminology Criteria for Adverse Events (CTCAE, version 5.0). In total, 35 eligible patients with unresectable HCC were included; 82.9% of patients had Hepatitis B virus (HBV) infection, and 88.6% of patients had liver cirrhosis. A total of 88.6% of patients had multiple tumors, and the median diameter of the largest tumor was 10.1 cm. A total of 14.3% of patients had extrahepatic metastasis, and 51.4% of patients had portal vein tumor thrombus. The percentages of BCLC stages A, B and C were 5.7%, 28.6% and 65.7%, respectively. There were 16 patients in the TACE + lenvatinib group and 19 patients in the TACE + lenvatinib + PD-1 group. The median follow-up time was 7.7 months (ranging from 1.7 to 31.6 months). Neither group reached the median overall survival. Under RECIST v1.1 criteria, the median PFS was 10.4 and 7.9 months in the TACE + lenvatinib and TACE + lenvatinib + PD-1 groups (HR, 1.13; 95% CI 0.45–2.84; p = 0.80), the objective response rates (ORR) were 31.3% and 31.6% (p > 0.05), and the disease control rates (DCR) were 93.8% and 78.9% (p > 0.05), respectively. Under mRECIST criteria, the median PFS was 10.4 and 10.1 months (HR, 0.98; 95% CI 0.38–2.54, p = 0.97), the ORR was 62.5% and 63.2% (p > 0.05), and the DCR was 93.8% and 73.7% (p > 0.05), respectively. Overall, AEs were relatively similar between the two groups. PD-1 inhibitors did not improve the PFS and tumor response of unresectable HCC treated with TACE plus lenvatinib. Hepatitis B infection, liver cirrhosis, portal vein tumor thrombus, multiple tumors and large tumor diameter may be potential factors that affect the efficacy of PD-1 inhibitors but need further validation.

## Introduction

Hepatocellular carcinoma (HCC) is the most common primary liver cancer and the second most frequent cause of cancer death worldwide^1^. Because of the occult symptoms of HCC, most patients are intermediate or advanced and unresectable when diagnosed^2^. For unresectable HCC, transarterial chemoembolization (TACE) and systemic treatment are recommended as the most commonly used therapeutic methods^1^.

TACE has become the standard of care for intermediate HCC, and it is also widely used in advanced HCC^3,4^. The efficacy and safety of TACE in the treatment of HCC has been supported by many meta‐analyses of randomized controlled trials^5,6^. The mechanism of TACE was the embolization of the tumor-feeding blood vessels and then the intra-arterial infusion of a cytotoxic agent, which resulted in strong cytotoxic and local hypoxia and ischemic necrosis targeted to the tumor^1^. This change in the local environment can lead to the activation of hypoxia-inducible factors (HIFs) and increased levels of vascular endothelial growth factor (VEGF), which could increase the efficacy of tyrosine-kinase inhibitors (TKIs) such as sorafenib and lenvatinib^7,8^. The TACTICS trial showed that TACE plus sorafenib significantly improved progression-free survival (PFS) (25.2 vs. 13.5 months; p = 0.006) and median time to untreatable (unTACEable) progression (26.7 vs. 20.6 months; p = 0.02) compared with TACE alone in patients with unresectable HCC^9^. Furthermore, lenvatinib, which was noninferior to sorafenib as validated in a phase III noninferiority trial, plus TACE has been confirmed to be more effective than lenvatinib monotherapy in improving the survival of advanced HCC patients with well-tolerated safety^10–13^. For those reasons, the combination of TACE and TKIs such as lenvatinib is currently widely used in clinical practice.

Programmed death receptor-1 (PD-1) inhibitors are key immunosuppressive transmembrane proteins expressed on the surface of T cells^14^. PD-1 inhibitors can bind to programmed death-ligand 1 (PD-L1), which is expressed in cancer cells, inhibit the function of T cells and reduce the tumor killing effect^15^. PD-1 inhibitors have been recognized as an effective antitumor therapy in the systemic treatment of advanced HCC^16^. Moreover, recent studies have shown that the combination of PD-1 inhibitors on the basis of TACE and lenvatinib may be the most promising combined treatment strategy for HCC. Since there is no high-level evidence supporting its validity, it is often questioned. Therefore, the primary purpose of our study was to explore whether PD-1 inhibitors would improve the prognosis of unresectable HCC treated with TACE plus lenvatinib.

## Materials and methods

### Patient cohorts

In this single-center retrospective study, the medical records were viewed to collect data on consecutive patients from March 2019 to June 2022. The inclusion criteria were as follows: (a) patients with HCC diagnosis by the European Association for the Study of the Liver criteria and aged ≥ 18 years old; (b) refractory to the standard first-line therapy (ablation, resection and transplant); (c) Child‒Pugh class A or B and Eastern Cooperation Oncology Group (ECOG) performance status of 0–1; (d) accept TACE, lenvatinib with or without PD-1 inhibitors for at least one complete cycle of treatment and assessment; and (e) without heart, lung or kidney dysfunction and life expectancy of ≥ 3 months (According to the Chinese clinical practice guidelines for TACE, patients with a life expectancy of less than 3 months are considered contraindications to TACE). The exclusion criteria were as follows: (a) acceptance of other antitumor treatments, such as PD-L1 inhibitors, hepatic arterial infusion chemotherapy (HAIC), radiotherapy and chemotherapy; (b) no measurable lesion by the Response Evaluation Criteria in Solid Tumors (RECIST v1.1 and mRECIST); and (c) incomplete clinical data and follow-up data.

This study was approved by the Institutional Ethics Committee of Mengchao Hepatobiliary Hospital of Fujian Medical University, and written informed consent was obtained from all study participants. All procedures were performed in accordance with the World Medical Association Declaration of Helsinki in 1964 and its later amendments or comparable ethical standards.

### Treatment

The TACE protocol was performed according to the Chinese clinical practice guidelines for TACE^17^. Briefly, using the Seldinger technique, the catheter sheath was retrogradely placed through the femoral artery. Using a digital subtraction angiography system, the catheter was inserted into the celiac trunk, superior mesenteric artery, and suspected tumor-supplying arteries to confirm their location, size, number and supply arteries of the tumor. Microcatheters were super-selected to each blood supply branch of the tumor and injected slowly with epirubicin mixed with iodine oil (not more than 30 ml of iodine oil was injected) until the iodine oil deposited well on the tumor lesion, the blood flow was slowed. Following that, gelatin sponge particles were injected into the microcatheter until completely stagnant. When tumor staining persisted after angiography, additional embolization was performed. In the end, the catheter guidewire was withdrawn, and the puncture site of the right femoral artery was bandaged with local pressure. Repeat TACE was based on the “on-demand” principle and depended on the judgment of the medical team. The initial use of lenvatinib or lenvatinib plus PD-1 inhibitor was within 7 days after the first TACE treatment. The lenvatinib dose was 8 mg (< 60 kg) or 12 mg (≥ 60 kg) once daily based on body weight. PD-1 inhibitors were administered intravenously at standard doses every 3 weeks, including camrelizumab, tislelizumab, sintilimab and pembrolizumab. Treatment-related adverse events (AEs) were evaluated according to the Common Terminology Criteria for Adverse Events (CTCAE, version 5.0). The dose of lenvatinib could be halved or continued after the symptoms of AEs were relieved after intermittent discontinuation. Dose adjustments were not allowed for PD-1 inhibitors.

### Follow-up and assessment

Follow-up was conducted every 4–6 weeks. Tumor response was assessed through contrast-enhanced CT or MRI according to the RECIST v1.1 and mRECIST criteria during follow-up, including complete response (CR), partial response (PR), stable disease (SD), and progressive disease (PD). The disease control rate (DCR) included CR, PR and SD. The objective response rate (ORR) included CR and PR. PFS and OS were calculated. OS was the time from the first TACE to death or the last follow-up. PFS was the time from first TACE to disease progression.

### Statistical analysis

Statistical analysis was performed using R (version 3.6.3). Categorical variables were expressed as frequencies with percentages and compared using the χ2 test or Fisher’s exact test. Continuous variables were expressed as the median [Q1, Q3] and compared using Student’s t test or the Mann‒Whitney U test. The survival curves of OS and PFS were analyzed using Kaplan–Meier analysis and the log rank test. p < 0.05 was considered statistically significant.

### Statement of ethics

This study was approved by the Institutional Ethics Committee of Mengchao Hepatobiliary Hospital of Fujian Medical University, and written informed consent was obtained from all study participants. All procedures were performed in accordance with the World Medical Association Declaration of Helsinki in 1964 and its later amendments or comparable ethical standards.

## Results

### Patient characteristics

**Figure 1:**
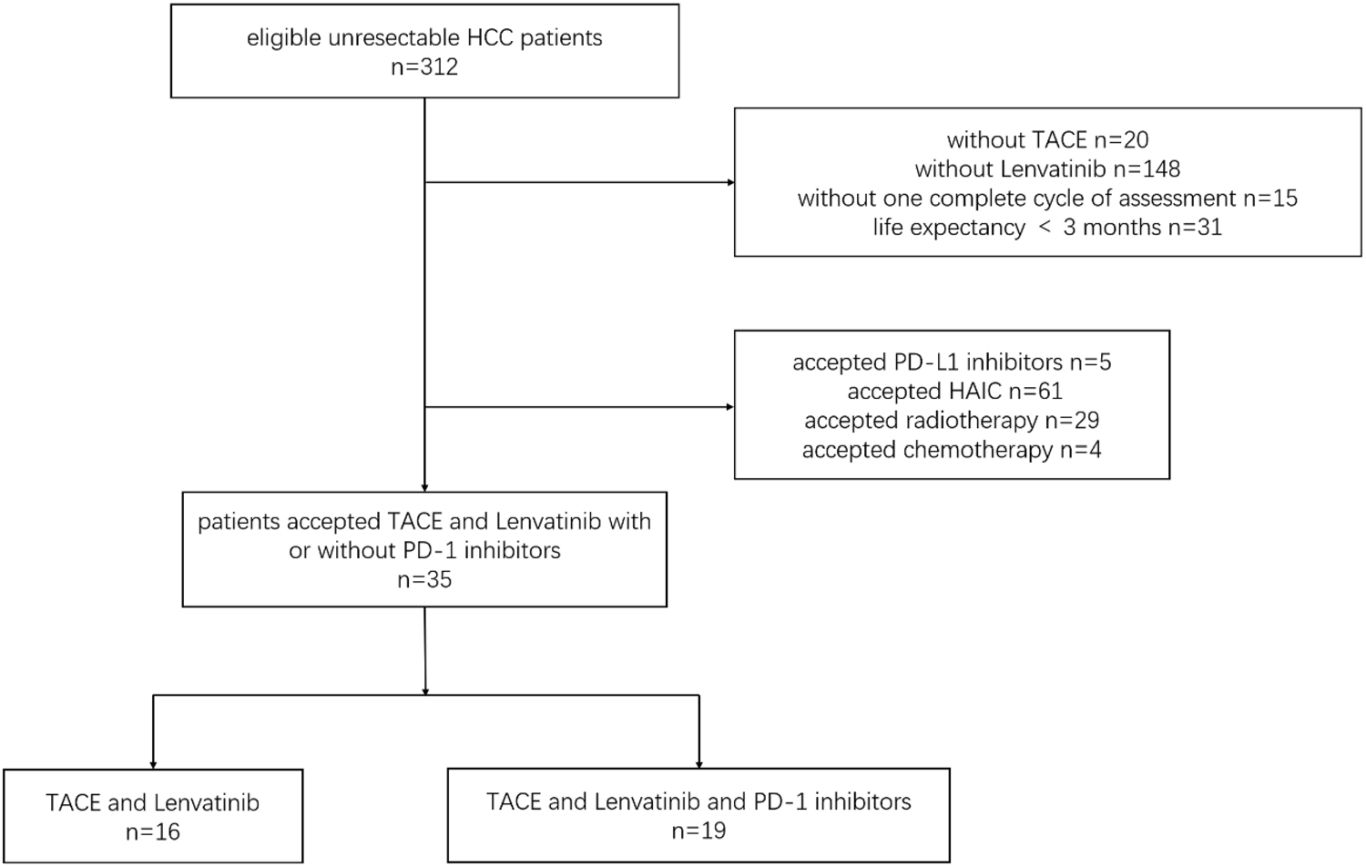
Flow diagram for enrolling patients in this study. HCC, Hepatocellular carcinoma; TACE, transarterial chemoembolization; HAIC, hepatic arterial infusion chemotherapy; PD-1, Programmed death receptor-1.

The flow diagram for enrolling patients is shown in Fig. 1. A total of 312 eligible unresectable HCC patients were screened. Finally, 35 patients were enrolled, 88.6% of whom were male, with a median age of 57 years. A total of 65.7% of patients were Child‒Pugh class A, and 91.4% of patients had an ECOG performance status of 1. A total of 82.9% of patients had HBV infection, and 88.6% of patients had liver cirrhosis. A total of 88.6% of patients had multiple tumors, and the median diameter of the largest tumor was 10.1 cm. A total of 14.3% of patients had extrahepatic metastasis, and 51.4% of patients had portal vein tumor thrombus. The percentages of BCLC stages A, B and C were 5.7%, 28.6% and 65.7%, respectively.

The TACE and lenvatinib group contained 16 patients, and the TACE and lenvatinib plus PD-1 inhibitor group contained 19 patients. The baseline patient characteristics were compared between the two groups, and the results are shown in Table 1. All the baseline patient characteristics were not significantly different between the two groups (p > 0.05).

**Table 1:** Baseline characteristics in two groups.

	Overall (N = 35)	TACE + Lenvatinib (N = 16)	TACE + Lenvatinib + PD-1 (N = 19)	p
Gender (n/%)				
Male	31 (88.6%)	13 (81.3%)	18 (94.7%)	0.474
Female	4 (11.4%)	3 (18.8%)	1 (5.3%)	
Age (years)				
Median [Q1, Q3]	57.0 [50.0, 65.0]	61.5 [53.0, 65.0]	57.0 [47.0, 64.5]	0.591
Child–Pugh class				
A	23 (65.7%)	10 (62.5%)	13 (68.4%)	0.992
B	12 (34.3%)	6 (37.5%)	6 (31.6%)	
ECOG performance				
0	3 (8.6%)	0 (0%)	3 (15.8%)	0.291
1	32 (91.4%)	16 (100%)	16 (84.2%)	
Platelet (× 10^9^/L)				
Median [Q1, Q3]	194 [121, 264]	167 [115, 230]	218 [124, 277]	0.190
Prothrombin time (s)				
Median [Q1, Q3]	13.5 [12.9, 14.3]	13.5 [12.8, 14.1]	13.9 [13.0, 14.4]	0.678
Albumin (g/dL)				
Median [Q1, Q3]	35.0 [32.5, 37.5]	36.5 [33.8, 37.3]	35.0 [32.0, 38.0]	0.551
Total bilirubin (µmol/L)				
Median [Q1, Q3]	16.7 [10.4, 24.4]	14.8 [10.0, 20.5]	17.8 [12.6, 28.6]	0.269
White blood cell (× 10^9^/L)				
Median [Q1, Q3]	6.11 [4.60, 7.15]	5.42 [4.54, 6.44]	6.21 [4.79, 7.85]	0.108
Hemoglobin (g/L)				
Median [Q1, Q3]	128 [108, 146]	118 [103, 135]	134 [118, 149]	0.052
Hepatitis B virus antigens				
Negative	6 (17.1%)	3 (18.8%)	3 (15.8%)	1.000
Positive	29 (82.9%)	13 (81.3%)	16 (84.2%)	
Serum HBV-DNA level (IU/mL)				
< 500	11 (31.4%)	5 (31.3%)	6 (31.6%)	1.000
≥ 500	24 (68.6%)	11 (68.8%)	13 (68.4%)	
Alpha fetoprotein (ng/mL)				
< 400	11 (31.4%)	3 (18.8%)	8 (42.1%)	0.264
≥ 400	24 (68.6%)	13 (81.3%)	11 (57.9%)	
PIVKA-II (mAU/ml)				
< 40	1 (2.9%)	1 (6.3%)	0 (0%)	0.930
≥ 40	34 (97.1%)	15 (93.8%)	19 (100%)	
Cirrhosis (n/%)				
No	4 (11.4%)	2 (12.5%)	2 (10.5%)	1.000
Yes	31 (88.6%)	14 (87.5%)	17 (89.5%)	
Number of tumors				
Single	4 (11.4%)	2 (12.5%)	2 (10.5%)	1.000
Multiple	31 (88.6%)	14 (87.5%)	17 (89.5%)	
Largest diameter of tumors (cm)				
Median [Q1, Q3]	10.1 [7.55, 13.1]	10.6 [7.13, 13.8]	10.0 [7.75, 12.5]	0.778
Extrahepatic metastasis				
No	30 (85.7%)	14 (87.5%)	16 (84.2%)	1.000
Yes	5 (14.3%)	2 (12.5%)	3 (15.8%)	
Portal vein tumor thrombus				
No	17 (48.6%)	8 (50.0%)	9 (47.4%)	1.000
Yes	18 (51.4%)	8 (50.0%)	10 (52.6%)	
BCLC stage				
A	2 (5.7%)	1 (6.3%)	1 (5.3%)	0.935
B	10 (28.6%)	5 (31.3%)	5 (26.3%)	
C	23 (65.7%)	10 (62.5%)	13 (68.4%)	

TACE: transarterial chemoembolization; PD-1: Programmed death receptor-1; ECOG: Eastern Cooperative Oncology Group; HBV: Hepatitis B virus; TACE: Transarterial chemoembolization; BCLC: Barcelona Clinic Liver Cancer; PIVKA-II: Serum protein induced by vitamin K absence or antagonist-II.

### Tumor response, prognosis and safety

The median follow-up time was 7.7 months (ranging from 1.7 to 31.6 months). The best tumor response of 35 patients in the two groups is shown in Table 2. Under the RECIST v1.1 criteria, the ORR were 31.3% in the TACE + lenvatinib group and 31.6% in the TACE + lenvatinib + PD-1 group, and the DCR were 93.8% and 78.9%, respectively. Under the mRECIST criteria, the ORR were 62.5%, and the DCR were 93.8% and 73.7%, respectively. Both ORR and DCR were not significantly different between the two groups according to the RECIST v1.1 and mRECIST criteria (p > 0.05).

**Table 2:** Tumor response in two groups.

	TACE + Lenvatinib (N = 16)	TACE + Lenvatinib + PD-1 (N = 19)	p
RECIST v1.1			
CR	0 (0%)	0 (0%)	
PR	5 (31.3%)	6 (31.6%)	
SD	10 (62.5%)	9 (47.4%)	
PD	1 (6.3%)	4 (21.1%)	
ORR	5 (31.3%)	6 (31.6%)	1.000
DCR	15 (93.8%)	15 ((78.9%)	0.446
mRECIST			
CR	0 (0%)	2 (10.5%)	
PR	10 (62.5%)	10 (52.6%)	
SD	5 (31.3%)	2 (10.5%)	
PD	1 (6.3%)	5 (26.3%)	
ORR	10 (62.5%)	12 (63.2%)	1.000
DCR	15 (93.8%)	14 (73.7%)	0.263

TACE: transarterial chemoembolization; PD-1: Programmed death receptor-1; RECIST: Response Evaluation Criteria in Solid Tumors; mRECIST: modified Response Evaluation Criteria in Solid Tumors; CR: complete response; PR: partial response; SD: stable disease; PD: progressive disease; ORR: Objective response rate; DCR: disease control rate.

**Figure 2:**
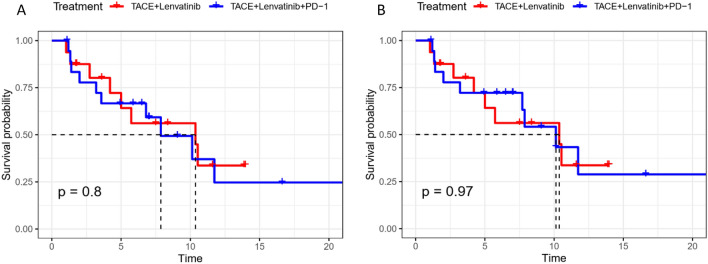
Kaplan–Meier analysis of median PFS in two groups under the RECIST v1.1 (**A**) and mRECIST (**B**) criteria. TACE, transarterial chemoembolization; PFS, progression-free survival.

The median OS was not reached in the two groups. No patient died in the TACE + Len group. In the TACE + lenvatinib + PD-1 group, one patient died due to liver failure caused by tumor progression. Under RECIST v1.1 criteria, the median PFS was 10.4 and 7.9 months in the TACE + lenvatinib and TACE + lenvatinib + PD-1 groups, respectively, and no significant difference was found between the two groups (HR, 1.13; 95% CI 0.45–2.84; p = 0.80). Under the mRECIST criteria, the median PFS was 10.4 and 10.1 months, respectively, and no significant difference was found between the two groups (HR, 0.98; 95% CI 0.38–2.54, p = 0.97). The Kaplan–Meier analysis of median PFS in the two groups under the RECIST v1.1 and mRECIST criteria is shown in Fig. 2.

In total, 32 patients (91.4%) experienced AEs of any grade, as shown in Table 3. A total of four patients, two in the TACE + lenvatinib group and two in the TACE + lenvatinib + PD-1 group, stopped treatment or changed treatment regimens because of intolerable AEs. Grade 3–4 AEs occurred in 7 patients (43.8%) in the TACE + lenvatinib group and in 9 patients (47.4%) in the TACE + lenvatinib + PD-1 group. The top five most common treatment-related AEs included fatigue, hypertension, hand-foot skin reaction, abnormal liver function, and hypothyroidism in the TACE + lenvatinib group, which was consistent with the TACE + lenvatinib + PD-1 group. No grade 5 AEs occurred in either group. Overall, AEs were relatively similar between the two groups.

**Table 3:** Adverse events in two groups.

Adverse events	TACE + Lenvatinib (N = 16)		TACE + Lenvatinib + PD-1 (N = 19)	
	Any grade	Grade 3–4	Any grade	Grade 3–4
Overall	15 (93.8%)	7 (43.8%)	17 (89.5%)	9 (47.4%)
Fatigue	10 (62.5%)	0 (0%)	13 (68.4%)	0 (0%)
Hypertension	9 (56.2%)	2 (12.5%)	13 (68.4%)	2 (10.5%)
Hand-foot skin reaction	9 (56.2%)	3 (18.8%)	12 (63.2%)	4 (21.1%)
Abnormal liver function	8 (50.0%)	2 (12.5%)	9 (47.4%)	3 (15.8%)
Hypothyroidism	6 (37.5%)	0 (0%)	6 (31.6%)	1 (5.3%)
Proteinuria	5 (31.3%)	0 (0%)	15 (78.9%)	0 (0%)
Decreased appetite	5 (31.3%)	0 (0%)	2 (10.5%)	0 (0%)
Hypothyroidism	4 (25.0%)	0 (0%)	10 (52.6%)	0 (0%)
Neutropenia	3 (18.8%)	0 (0%)	4 (21.1%)	0 ((0%)
Dysphonia	2 (12.5%)	0 (0%)	2 (10.5%)	0 (0%)
Arthralgia	1 (6.3%)	0 (0%)	5 (26.3%)	0 (0%)
Ventosity	1 (6.3%)	0 (0%)	12 (63.2%)	0 (0%)
Abdominal pain	1 (6.3%)	0 (0%)	14 (73.7%)	0 (0%)

TACE: transarterial chemoembolization; PD-1: Programmed death receptor-1;

## Discussion

Our retrospective study found that PD-1 inhibitors plus TACE and lenvatinib did not improve PFS compared with TACE plus lenvatinib, and there was no significant difference in tumor response according to either the RECIST v1.1 or mRECIST criteria. The results suggest that the addition of PD-1 inhibitors to TACE and lenvatinib treatment did not improve the first-line treatment of unresectable HCC.

The optimal treatment strategy for unresectable HCC remains undefined in real-world practice because of the highly heterogeneous nature of HCC populations. TACE and lenvatinib are effective combination treatments for unresectable HCC. A propensity score matching (PSM) study enrolled 38 intermediate-stage HCC patients to evaluate the efficacy and safety of lenvatinib-TACE sequential therapy. The mPFS was 11.6 months with well-tolerated safety, and the ORR was 63.2% under mRECIST criteria^12^. LAUNCH was a Phase III, randomized clinical trial; the mPFS was 10.6 months in the lenvatinib-TACE group, and the ORR was 54.1% according to the mRECIST criteria^18^. Our results showed that the ORR was 62.5% in the TACE + lenvatinib group, and the mPFS was 10.4 months under mRECIST criteria. The results were similar to the reported studies above, and we verified the excellent performance of TACE + lenvatinib in clinical practice.

Although PD-1 inhibitors have shown promising anticancer activity in several carcinomas, their application in HCC remains controversial, especially in first-line treatment. In monotherapy, the CheckMate459 trial showed that first-line nivolumab treatment did not significantly improve PFS compared with sorafenib (median PFS, 3.8 vs. 3.9 months, HR = 0.98; 95% CI 0.82–1.18)^19^. In combination therapy, the LEAP-002 trial showed that lenvatinib plus pembrolizumab versus lenvatinib in advanced or unresectable HCC failed to reach the primary endpoint (median PFS 8.3 vs. 8.1 months, HR, 0.834; 95% CI 0.712–0.978)^20^. The two clinical trials did not support the application of nivolumab or pembrolizumab in first-line treatment, indicating that PD-1 inhibitors need further verification in first-line treatment.

Recently, some studies have reported the effectiveness of TACE, lenvatinib and PD-1 inhibitor triple therapy in unresectable HCC. Yan et al. reported a multicenter retrospective study that enrolled 62 patients with unresectable HCC who were administered lenvatinib and PD-1 inhibitors and underwent TACE; the ORR was 77.4%, and 53.2% of patients were transformed to resectable HCC^21^. Wang et al. found that TACE combined with lenvatinib and PD-1 inhibitors was superior to TACE plus lenvatinib or TACE alone; the mPFS was 24.1 months, and the best objective response rate and best disease control rate were 70.4% and 100.0%, respectively, based on the mRECIST criteria^22^. Zou et al. found that TACE-lenvatinib-PD-1 treatment can improve the survival of patients with HCC and portal vein tumor thrombus compared with TACE-lenvatinib (mOS 23.5 vs. 18.3 months, mPFS 7.5 vs. 4.3 months, ORR 38.57% vs. 24.45%, DCR 80.00% vs. 56.67%, p < 0.05, based on the mRECIST criteria)^23^. Cai et al. reported a similar positive result (mOS 16.9 vs. 12.1 months, mPFS 7.3 vs. 4.0 months, ORR 56.1% vs. 32.5%, DCR 85.4% vs. 62.5%, p < 0.05, based on the mRECIST criteria)^24^. The above results have all achieved positive results, indicating that PD-1 inhibitors can improve the prognosis of patients with TACE and lenvatinib. However, in contrast, we achieved a negative result, and the addition of PD-1 inhibitors resulted in shorter PFS and lower tumor response. The difference might be attributed to the worse baseline characteristics. First, in our study, 34.3% of patients had Child‒Pugh class B cirrhosis, and 88.6% of patients had a history of cirrhosis. This proportion is higher than that in any of the above studies, indicating that a large proportion of HCC patients have impaired liver function. It has been proven that insufficient liver function reserve was associated with poor OS and PFS in unresectable HCC treated with lenvatinib plus anti-PD-1 antibody^25–27^. Second, the percent of hepatitis B infection was as high as 82.9%, and 68.6% of patients had serum HBV DNA levels ≥ 500 IU/mL in our cohorts. Although some studies shown that TACE and lenvatinib plus PD-1 inhibitors could achieve positive or optimistic results, the patients with high HBV-DNA load in their cohort are far lower than us: HBV‐DNA ≥ 50 IU/mL accounts for 7.9% in 139 patients in Wang’s study^28^, HBV‐DNA ≥ 2000 copy/mL accounts for 34.5% in 55 patients in Wu’s study^29^, HBV‐DNA ≥ 1000 copy/mL accounts for 54.8% in 62 patients in Wu’s another study^21^. Previous studies have shown that HBV-related HCC microenvironment is more immunosuppressive and exhausted. More direct evidence suggests that HBV could induce immune cell dysfunction and a decline in immune cell quantity, disrupting PD-1/PD-L1 blockade efficacy in HBV HCC^30^. Therefore, we consider that High HBV-DNA load may impact the effectiveness of PD-1 inhibitors. Third, more than half of the patients had portal vein tumor thrombus (PVTT) (51.4% overall), the median diameter was 10.1 cm, and 88.6% of patients had multiple tumors, indicating the great tumor burden of these patients. PVTT, large tumors and multiple tumors were proven to be significant prognostic factors associated with worse survival^31,32^. Typically, the treatment efficiency was not satisfactory in HCC with those factors. Therefore, blindly using PD-1 inhibitors may not benefit those patients and brings a great economic burden. We should re-examine the efficacy of triple therapy (PD-1 inhibitors + TACE + lenvatinib) unless there is higher-level medical evidence in the future.

Our study has several limitations. The first limitation was our sample size. Subgroup analysis could not be conducted due to the limited sample size, and it was not possible to determine whether there were any populations that could potentially benefit from the treatment more than others. Second, the single-center retrospective study design might lead to potential selection bias, and the follow-up time was relatively short. Third, due to individual differences in patients, the dosage, frequency, and interval time of TACE cannot be consistent, which may affect the efficacy. Notably, publication bias may exist in previous retrospective studies with small sample sizes that achieved positive results. Therefore, it is necessary to conduct a multicenter randomized controlled trial (RCT) with more cases in the future to validate the efficiency of triple therapy, including TACE, and lenvatinib and PD-1 inhibitor administration, in unresectable HCC patients.

## Conclusion

PD-1 inhibitors did not improve the PFS and tumor response of unresectable HCC treated with TACE plus lenvatinib. Hepatitis B infection, liver cirrhosis, portal vein tumor thrombus, multiple tumors and large tumor diameter may be potential factors that affect the efficacy of PD-1 inhibitors but need further validation.

## Data Availability

The datasets used and/or analyzed during the current study are available from the corresponding author on reasonable request.

## References

[1] European Association for the Study of the Liver (2018). Electronic address, e.e.e., &L. European Association for the Study of the, EASL Clinical Practice Guidelines: Management of hepatocellular carcinoma. J. Hepatol..

[2] Forner A, Reig M, Bruix J (2018). Hepatocellular carcinoma. Lancet.

[3] Llovet JM, Kelley RK, Villanueva A, Singal AG, Pikarsky E, Roayaie S (2021). Hepatocellular carcinoma. Nat. Rev. Dis. Primers.

[4] Zhou J, Sun H, Wang Z, Cong W, Wang J, Zeng M (2020). Guidelines for the diagnosis and treatment of hepatocellular carcinoma (2019 edition). Liver Cancer..

[5] Lencioni R, de Baere T, Soulen MC, Rilling WS, Geschwind JF (2016). Lipiodol transarterial chemoembolization for hepatocellular carcinoma: A systematic review of efficacy and safety data. Hepatology.

[6] Llovet JM, Bruix J (2003). Systematic review of randomized trials for unresectable hepatocellular carcinoma: Chemoembolization improves survival. Hepatology.

[7] Li X, Feng GS, Zheng CS, Zhuo CK, Liu X (2004). Expression of plasma vascular endothelial growth factor in patients with hepatocellular carcinoma and effect of transcatheter arterial chemoembolization therapy on plasma vascular endothelial growth factor level. World J. Gastroenterol..

[8] Teng Y, Ding X, Li W, Sun W, Chen J (2022). A retrospective study on therapeutic efficacy of transarterial chemoembolization combined with immune checkpoint inhibitors plus lenvatinib in patients with unresectable hepatocellular carcinoma. Technol. Cancer Res. Treat..

[9] Kudo M, Ueshima K, Ikeda M, Torimura T, Tanabe N, Aikata H (2020). Randomised, multicentre prospective trial of transarterial chemoembolisation (TACE) plus sorafenib as compared with TACE alone in patients with hepatocellular carcinoma: TACTICS trial. Gut.

[10] Kudo M, Finn RS, Qin S, Han KH, Ikeda K, Piscaglia F (2018). Lenvatinib versus sorafenib in first-line treatment of patients with unresectable hepatocellular carcinoma: A randomised phase 3 non-inferiority trial. Lancet.

[11] Xia D, Bai W, Wang E, Li J, Chen X, Wang Z (2022). Lenvatinib with or without concurrent drug-eluting beads transarterial chemoembolization in patients with unresectable, advanced hepatocellular carcinoma: a real-world, multicentre, retrospective study. Liver Cancer.

[12] Ando Y, Kawaoka T, Amioka K, Naruto K, Ogawa Y, Yoshikawa Y (2021). Efficacy and safety of lenvatinib-transcatheter arterial chemoembolization sequential therapy for patients with intermediate-stage hepatocellular carcinoma. Oncology.

[13] Fu Z, Li X, Zhong J, Chen X, Cao K, Ding N (2021). Lenvatinib in combination with transarterial chemoembolization for treatment of unresectable hepatocellular carcinoma (uHCC): A retrospective controlled study. Hepatol. Int..

[14] Blank C, Gajewski TF, Mackensen A (2005). Interaction of PD-L1 on tumor cells with PD-1 on tumor-specific T cells as a mechanism of immune evasion: Implications for tumor immunotherapy. Cancer Immunol. Immunother..

[15] Cha JH, Chan LC, Li CW, Hsu JL, Hung MC (2019). Mechanisms controlling PD-L1 expression in cancer. Mol. Cell.

[16] Sun LY, Zhang KJ, Xie YM, Liu JW, Xiao ZQ (2023). Immunotherapies for advanced hepatocellular carcinoma. Front. Pharmacol..

[17] Clinical Guidelines Committee of Chinese Interventionalists, C. (2021) Chinese clinical practice guidelines for transarterial chemoembolization of hepatocellular carcinoma. *Zhonghua Nei Ke Za Zhi*. **60**(7), 599–614. 10.3760/cma.j.cn112137-20210425-00991 34619836

[18] Peng Z, Fan W, Zhu B, Wang G, Sun J, Xiao C (2023). Lenvatinib combined with transarterial chemoembolization as first-line treatment for advanced hepatocellular carcinoma: A phase III, randomized clinical trial (LAUNCH). J. Clin. Oncol..

[19] Yau T, Park JW, Finn RS, Cheng AL, Mathurin P, Edeline J (2022). Nivolumab versus sorafenib in advanced hepatocellular carcinoma (CheckMate 459): A randomised, multicentre, open-label, phase 3 trial. Lancet Oncol..

[20] Finn RS, Kudo M, Merle P, Meyer T, Qin S, Ikeda M (2022). LBA34 Primary results from the phase III LEAP-002 study: Lenvatinib plus pembrolizumab versus lenvatinib as first-line (1L) therapy for advanced hepatocellular carcinoma (aHCC). Ann. Oncol..

[21] Wu JY, Yin ZY, Bai YN, Chen YF, Zhou SQ, Wang SJ (2021). Lenvatinib combined with anti-PD-1 antibodies plus transcatheter arterial chemoembolization for unresectable hepatocellular carcinoma: A multicenter retrospective study. J. Hepatocell. Carcinoma.

[22] Wang, W.J., Z.H. Liu, K. Wang, H.M. Yu, Y.Q. Cheng, Y.J. Xiang, *et al*. Efficacy and safety of TACE combined with lenvatinib and PD-1 inhibitors for unresectable recurrent HCC: A multicenter, retrospective study. *Cancer Med*. (2023). 10.1002/cam4.5880 PMC10242311 36999793

[23] Zou, X., Q. Xu, R. You, &G. Yin. Correlation and efficacy of TACE combined with lenvatinib plus PD-1 inhibitor in the treatment of hepatocellular carcinoma with portal vein tumor thrombus based on immunological features. *Cancer Med*. (2023). 10.1002/cam4.5841 PMC10242346 36951443

[24] Cai M, Huang W, Huang J, Shi W, Guo Y, Liang L (2022). Transarterial chemoembolization combined with lenvatinib plus PD-1 inhibitor for advanced hepatocellular carcinoma: A retrospective cohort study. Front. Immunol..

[25] Xu MH, Huang C, Li ML, Zhu XD, Tan CJ, Zhou J (2023). Effectiveness and safety of lenvatinib plus anti-programmed death-1 antibodies in patients with hepatocellular carcinoma: A real-world cohort study. Cancer Med..

[26] Yao J, Zhu X, Wu Z, Wei Q, Cai Y, Zheng Y (2022). Efficacy and safety of PD-1 inhibitor combined with antiangiogenic therapy for unresectable hepatocellular carcinoma: A multicenter retrospective study. Cancer Med..

[27] Scheiner B, Kirstein MM, Hucke F, Finkelmeier F, Schulze K, von Felden J (2019). Programmed cell death protein-1 (PD-1)-targeted immunotherapy in advanced hepatocellular carcinoma: Efficacy and safety data from an international multicentre real-world cohort. Aliment. Pharmacol. Ther..

[28] Wang WJ (2023). Efficacy and safety of TACE combined with lenvatinib and PD-1 inhibitors for unresectable recurrent HCC: A multicenter, retrospective study. Cancer Med..

[29] Wu XK (2024). Transcatheter arterial chemoembolisation combined with lenvatinib plus camrelizumab as conversion therapy for unresectable hepatocellular carcinoma: A single-arm, multicentre, prospective study. EClinicalMedicine.

[30] Li B, Yan C, Zhu J, Chen X, Fu Q, Zhang H (2020). Anti-PD-1/PD-L1 blockade immunotherapy employed in treating hepatitis B virus infection-related advanced hepatocellular carcinoma: A literature review. Front. Immunol..

[31] Xia WL, Zhao XH, Guo Y, Cao GS, Wu G, Fan WJ (2022). Transarterial chemoembolization combined with apatinib with or without PD-1 inhibitors in BCLC stage C hepatocellular carcinoma: A multicenter retrospective study. Front. Oncol..

[32] Li S, Wu J, Wu J, Fu Y, Zeng Z, Li Y (2023). Prediction of early treatment response to the combination therapy of TACE plus lenvatinib and anti-PD-1 antibody immunotherapy for unresectable hepatocellular carcinoma: Multicenter retrospective study. Front. Immunol..

